# The Effect of Carbamazepine on Performance, Carcass Value, Hematological and Biochemical Blood Parameters, and Detection of Carbamazepine and Its Metabolites in Tissues, Internal Organs, and Body Fluids in Growing Rabbits

**DOI:** 10.3390/ani13122041

**Published:** 2023-06-20

**Authors:** Lukáš Zita, Sebnem Kurhan, Ondřej Krunt, Eva Chmelíková, Adam Kraus, Jaroslav Čítek, Pavel Klouček, Roman Stupka

**Affiliations:** 1 Department of Animal Science, Faculty of Agrobiology, Food and Natural Resources, Czech University of Life Sciences Prague, 165 00 Prague, Czech Republic; krunt@af.czu.cz (O.K.);; 2 Department of Food Science, Faculty of Agrobiology, Food and Natural Resources, Czech University of Life Sciences Prague, 165 00 Prague, Czech Republic; 3 Department of Veterinary Sciences, Faculty of Agrobiology, Food and Natural Resources, Czech University of Life Sciences Prague, 165 00 Prague, Czech Republic; chmelikova@af.czu.cz

**Keywords:** carbamazepine, carbamazepine-10,11-epoxide, *trans*-10,11-dihydro-10,11-dihydroxy-CBZ, metabolite, rabbit, growth, feed, blood, internal organs, liver

## Abstract

**Simple Summary:**

Carbamazepine (CBZ) is one of the most prescribed antiepileptic drugs, which is prescribed as the first-line drug for the treatment of partial and generalized tonic–clonic epileptic seizures and psychosis. CBZ is among the three most frequently detected and most affected pharmaceutical residues in aquatic ecosystems. The objective of the study was to evaluate the effect of CBZ doses on rabbits’ productive performance, health, and the distribution of CBZ in animal tissues. The purpose of the study was to evaluate if CBZ or its metabolites would remain in meat or internal organs or if it would affect the animals in terms of growth or health. Productive performance was not affected by CBZ. On the other hand, CBZ doses reduced white and red blood cells. Most of the CBZ was excreted in feces, while the distribution to rabbit meat was low. Based on the results, there should not be any worries about CBZ intake from meat which was exposed to CBZ when the animals were alive.

**Abstract:**

Antiepileptic drugs (e.g., carbamazepine; CBZ) are widely prescribed for various conditions beyond epilepsy, including neurologic and psychiatric disorders. These medications can have both favorable and unfavorable impacts on mood, anxiety, depression, and psychosis. CBZ has been found at low concentrations (in the unit of nanograms per liter) in rivers, surface water, and even drinking water. As a result, when reclaimed wastewater is used for irrigation in agricultural ecosystems, CBZ can be reintroduced into the environment. That is why we tested different doses of CBZ in rabbits’ feed as the meat is consumed in every community, has no religious barriers, and the potential risk of consuming meat which has been exposed to CBZ treatment is not known. Also, the evidence of the effect of CBZ on rabbits is missing. Mainly, the CBZ doses affected the count of leukocytes and other blood traits, meaning the higher the dose, the higher the reduction. Moreover, there were only low amounts of CBZ in rabbits’ meat or tissues when they were exposed to the treatment.

## 1. Introduction

Carbamazepine (CBZ) is one of the most commonly prescribed antiepileptic drugs, which is prescribed as a first-line drug for the treatment of partial and generalized tonic–clonic epileptic seizures and psychosis [1,2]. CBZ is among the three most frequently detected and most affected pharmaceutical residues in aquatic ecosystems [3]. CBZ was first synthesized in the early 1950s [4]. It was introduced in Europe by Bonduelle in 1964 as an antiepileptic drug [5]. Antiepileptic drugs (AEDs) are used extensively to treat multiple non-epilepsy disorders, both in neurology and psychiatry [6]. The AEDs commonly have both positive and negative effects on mood, anxiety, depression, and psychosis [7]. CBZ is widely used as a drug for treating humans, as it is known to have an effect leading to mood improvements [8]. Bertilsson [9] observed the effectiveness of CBZ to be as functional as the drug phenytoin in the treatment of grand mat and psychomotor epilepsy. He also noted that CBZ is the first-choice drug in trigeminal neuralgia. The therapeutic anticonvulsant mechanism is primarily related to the blockade of the presynaptic voltage-gated sodium channels. The blockade of the sodium channels is believed to inhibit the release of synaptic glutamate and possibly other neurotransmitters [10]. The prescribed dosages to most patients range between 400 and 1200 mg per day, resulting in CBZ plasma levels of 4–12 μg∙mL^−1^ [11]. Nowadays, CBZ is generally used to treat seizure disorders and neuropathic pain. Moreover, it has a significant off-label use for the second-line treatment of bipolar disorder and used in combination with antipsychotics in some cases of schizophrenia when treatment with conventional antipsychotics alone has failed. It is also used when attention deficit hyperactivity disorder, schizophrenia, phantom limb syndrome, complex regional pain syndrome, borderline personality disorder, and post-traumatic stress disorder appear [12]. CBZ is ubiquitous in effluents and persistent during wastewater treatment [10]. Low levels (ng∙L^−1^) of CBZ were also reported in rivers and surface water and even in drinking water [10,13] Thus, it is reintroduced into agricultural ecosystems upon irrigation with reclaimed wastewater. People consuming produce irrigated with reclaimed wastewater were found to be exposed to CBZ. This was confirmed by the study of Paltiel et al. [14], who demonstrated that healthy individuals consuming reclaimed wastewater-irrigated (100 μg∙L^−1^ CBZ) produce excreted carbamazepine and its metabolites in their urine, while subjects consuming fresh-water-irrigated produce excreted undetectable or significantly lower levels of carbamazepine.

Response effects of CBZ were reported in fish [15], poultry [16], rats [7], and rabbits [17]. In fish, chronic exposure to environmental relevant levels of CBZ impacted multiple organs [15]. For example, exposure to CBZ led to a dose-dependent decline in reproduction and increase in internal organ pathologies in adult fish. Behavioral tests also demonstrated altered responses of fish to environmental stimuli that were correlated with gradual increases in environmental CBZ dosages [13]. Moreover, in poultry, during the incubation of Lohmann eggs, Kohl et al. [16] added a CBZ solution at quantitates of 0.02, 0.2, 2, 20, and 200 pg∙mg^−1^ embryonic weight to the chicken embryo to study the effects of CBZ. (These CBZ concentrations correspond to concentrations of 0.01, 0.1, 1, 10, 100 μg∙L^−1^, respectively, which are reported in treated wastewater). The authors indicated that exposure to 0.2 pg∙mg^−1^ CBZ resulted in a marked increase in abnormal or dead embryos. Moreover, the higher CBZ concentration (2–200 pg∙mg^−1^) resulted in a dose-dependent increase in embryonic mortality (from 25 to 65%) and a decrease in mal-formed or growth-delayed embryos (from 50 to 30%). In the study of Zimcikova et al. [7], the effect of CBZ on rats was observed; the authors reported that a dose of 28 mg∙25 g^−1^ standard laboratory diet (Carbamazepinum, G.L. Pharma GmbH) for 12 weeks significantly reduced the body weight compared to the control group with a blood serum drug concentration of 12 µmol∙L^−1^. Moreover, the number of fecal pellets was significantly increased in the carbamazepine group compared to the control one. However, no significant effect was detected in the rat’s behavior. Sirlak et al. [17] reported that a single dose of 20 mg∙kg^−1^ per day of CBZ in rabbits (Tegretol oral suspension, 100 mg∙5 mL^−1^; Novartis Pharmaceuticals, Basel, Switzerland) was administered by oral gavage for 5 days before the experiment. The authors indicated that the CBZ group showed ganglion cells with normal nuclei and cytoplasm leading to the conclusion that CBZ may protect the spinal cord from ischemic reperfusion injury that is associated with ameliorated neurologic and histopathologic results.

The study aimed to investigate the effect of the different sizes of carbamazepine (CBZ) doses on productive parameters, carcass traits, blood profile, and determination (detection) of CBZ and its metabolites in tissues, internal organs, and body fluids in growing rabbits as a potential risk.

## 2. Materials and Methods

### 2.1. Animals and Experimental Design

A total of 36 rabbits of the commercial Hyplus cross-bred line (PS 19 × PS 40), of both sexes (1:1), were weaned at 35 d of age and randomly divided into one control and two experimental groups (Table 1).

Rabbits were kept individually in the metabolic cage (0.15 m^2^ per rabbit). The cages (stainless steel) were constructed from a 3 mm diameter mesh floor and mesh walls. Solid feces were separated on a perforated tray under the cage and the urine drained into a collection container. These rabbit cages conform to current legislation in the Czech Republic and the requirements of the Directive of the European Parliament and of the Council 2010/63/EC, which legislates the requirements on the external environment of experimental animals.

The rabbits were housed in an area with controlled environmental conditions. The ventilation and control system allowed an ambient temperature to be maintained between 18 and 21 °C, and a relative humidity between 65 and 70%. The daily light period used was 12 h. All rabbits were fed ad libitum by a feeder with a control pelleted diet (14.5% crude protein, 10.2 MJ of digestible energy∙kg^−1^) and had ad libitum access to water from nipple drinkers. Ingredient and chemical composition of the control diet was according to Krunt et al. [18].

From the 35th day of age (adaptation period) the rabbits were fed only the control feed mixture ad libitum. From the 42nd day of age, the control group (control) was fed with the control feed mixture, without the addition of CBZ (Carbamazepine 5*H*-Dibenz[*b,f*]azepine-5-carboxamide; Merck KGaA, Darmstadt, Germany) ad libitum. Rabbits from the experimental group (CBZ-low dose) obtained 10 g of feed mixture (0.1 mg CBZ) per 1 kg of live weight (the dose increased linearly with the live weight of the rabbits, methodology based on animal or human dosing) and after they had consumed this dose, they obtained a control feed mixture, which they had full access to for the rest of the day. The experimental group (CBZ-high dose) was exposed to the same treatment as the previous group, but with a different dose, which was 12.5 mg CBZ in 10 g of feed mixture per day for 1 kg of live weight.

The amount of feed mixture with CBZ was based on the live weight of the rabbits, which was determined every day between 8 and 9 a.m. All rabbits from all groups, including the control group, were weighed to eliminate the effect of weighing and handling the rabbits.

### 2.2. Growth and Slaughter Performance, Physical Characteristics

Individual live weight was measured every day (between 8 and 9 a.m.) to determine the weight of the experimental feed mixture. Individual live weight was recorded weekly to determine growth parameters; feed intake was noted once per day, to calculate daily body weight gain, daily feed consumption and the feed conversion ratio.

All rabbits were weighed (SW) at the end of the experiment (77 d of age) and slaughtered. Slaughter and carcass dissection progresses were evaluated in accordance with the norms of the World Rabbit Science Association recommendation by Blasco and Ouhayoun [19]. Twenty minutes after slaughter, carcasses with the head, liver, kidneys, perirenal and scapular fat, and sets of organs consisting of thymus, trachea, esophagus, lungs, and heart were weighed (hot carcass; HC). After cooling for 24 h at 4 °C, the carcasses were weighed to obtain the chilled carcasses (CC). The cooler carcass shrinkage (CCS) was calculated as the difference between HC and CC, relative to the HC. The head, liver, kidneys, and set of organs were removed from each carcass to obtain the reference carcass (RC), which included the meat, bones, and fat deposits. The dressing percentage (DP; CC divided by SW and multiplied by 100) and the liver, kidneys, and hind leg weights were expressed as a percentage of the CC.

The pH of the loin and thigh (when referring to the loin and thigh in the text, it was specifically *longissimus lumborum* and *biceps femoris* muscles, which were taken into account with analyses) was measured 24 h post mortem (pHu). pH meter WTW pH 330i (WTW, Weilheim, Germany) was provided with a glass electrode suitable for meat penetration.

### 2.3. Blood Analyses

Blood samples from 12 animals from each group were taken during slaughtering at the age of 77 d from the jugular vein and were the subject of haematological and biochemical examination. Two samples of blood were obtained from each rabbit, one was contained K_2_EDTA for haematological analysis and CBZ determination, and one for biochemical analysis with no K_2_EDTA, which was collected into plastic vacutainer tubes. The samples with blood serum for biochemical tests were centrifuged at 1000× *g* for 10 min and were stored at −80 °C until analysis.

Haematological parameters were analyzed within 24 h after sample collection; before analysis, the samples were stored at 4 °C. The automatic haematological analyzer Coul-ter model ZF (Coulter Electronics, LTD., High Wycombe, UK) was used for the analysis. Erythrocytes, number of leukocytes and platelets, haemoglobin concentration, and haematocrit were determined. Biochemical indicators in serum such as the total protein, albumin, globulin, alanine aminotransferase (ALT), alkaline phosphatase (ALP), amylase, aspartate aminotransferase (AST), glutamate oxaloacetate transaminase (GOT), glutamate pyruvate transaminase (GPT), gamma glutamyl transferase (GGT), creatinine, glucose, urea, triacylglycerol, cholesterol, calcium, phosphorus, chlorine, magnesium, potassium, and sodium were photometrically measured in an automatic analyzer XL—200 (Erba Lachema, s.r.o., Brno, Czech Republic) by using a standard commercial kit (Erba Lachema, s.r.o., Czech Republic).

### 2.4. Chemical Analyses—Determination of CBZ and Metabolites

Samples for analysis (liver, kidney, content of the caecum, spleen, loin and thigh meat, hard feces) were taken during slaughter and slaughter dissection and subsequently individually packed in plastic bags. Liquid samples (blood, urine) were collected in test tubes. All samples for the determination of the level of CBZ metabolites were frozen at −80 °C until analysis. Prior to analysis, tissues were freeze-dried and ground. An aliquot of 1 g or 0.1 g freeze-dried representative sample for each tissue group was used in the residue analysis.

#### 2.4.1. Analytical Chemicals and Stock Solutions

The analytical standard CBZ was purchased from Merck KGaA (Darmstadt, Germany), *trans*-10,11-dihydro-10-hydroxyCBZ (CBZ-diol) and carbamazepine-10,11-epoxide (CBZ-E) were from Cerilliant. Isotopically labelled ^13^C_6_-carbamazepine (Cerilliant Corporation, Round Rock, TX, USA) and carbamazepine-10,11-epoxide-d_10_ (Santa Cruz Biotechnology, Huissen, The Netherlands) were used as internal standards (ISTD).

LC-MS grade acetonitrile (MeCN) (Honeywell, Leuven, Belgium) was used as an extraction solvent. Ultrapure water was obtained from MiliQ distilled water system and used for sample rehydration and mobile phase (A) with 0.1% formic acid and ammonium formate at 5 mM in water (99%, LiChropur, Merck KGaA, Darmstadt, Germany) was used.

Extraction was performed using an EN 15,662 QuEChERS (Bond Elute, Agilent, Santa Clara, CA, USA) salt mixture. Each pouch contained 0.5 g of disodium citrate sesquihydrate, 1 g of sodium citrate, 1 g of sodium chloride, and 4 g of magnesium sulphate. An aliquot of 650 mg and 300 mg QuEChERS salt mix were portioned into the microcentrifuge tubes.

QuEChERS extracts were further cleaned up with the AOAC method dispersive solid phase extraction (dSPE) kit when available. Otherwise, each individual dSPE tube was prepared by weighing 150 mg of MgSO_4_ (Honeywell, Leuven, Belgium), 50 mg of primary secondary amine (PSA, Agilent Technologies, Santa Clara, CA, USA), and 50 mg of C18 (DSC-18 SPE, Merck KGaA, Darmstadt, Germany) into a 2 mL microcentrifuge tube. MgSO_4_ was dried in an oven overnight at 250 °C before being incorporated into the dSPE tubes. PSA (25 mg) was used to clean up urine samples.

Stock solutions of internal standards, parent drug (CBZ), and its metabolites were prepared individually in MeCN. Each internal standard solution was combined to achieve 1 µg∙mL^−1^ working concentration. Parent drug (CBZ) and metabolites were also mixed in MeCN at 1 µg∙mL^−1^ to be used in calibration and validation.

#### 2.4.2. Validation

Liver and kidney extraction methods were validated using blank pork liver and kidney obtained from a local store. Identical pre-treatments (lyophilization, grinding) were applied to pork liver and kidney samples. Samples were spiked with the CBZ and metabolite cocktail to achieve final concentrations of 0.5, 1.0 and 10 ng∙g^−1^. Linearity (sensitivity) of the calibration range was checked from eight concentration levels (0.03, 0.1, 0.3, 1.0, 3.3, 10.0, 33.3, 100.0 ng∙mL^−1^). Trueness (Recovery%) was calculated based on the spiked blank sample’s practical concentration to theoretical concentration (70–120%) ratio. Precision (repeatability) was calculated as the % relative standards deviation (RSD%) of the same level spiked samples. The lowest spiking level was stated as the limit of quantification (LOQ) when the trueness (70–120%) and RSD% (<20%) met the acceptance criteria. Validation was performed using three replicates of each tissue and concentration. Previously developed and validated urine and blood extraction methods for rabbits were used in this experiment.

#### 2.4.3. Extraction

##### Extraction of Liver, Kidney, and Thigh Samples

Each freeze-dried solid sample (1 g) was weigh into 50 mL centrifuge tube. An aliquot of 25 µL internal standard (1µg∙mL^−1^) mixture was pipetted onto the sample and mixed. Before extraction, samples were rehydrated with 10 mL of water and left to stand at 4 °C for 30 min. In order to extract the target analytes, 10 mL of MeCN was transferred onto the sample and the tube content was vortexed for 3 × 20 s and QuEChERS salt pouch (6.5 g) was added to the content, vortexed to avoid clumping, and 6 mL of MeCN extract was collected in a clean 15 mL tube after centrifugation (7806 RCF, +4 °C, 10 min). An aliquot of 6 mL n-hexane was pipetted onto the MeCN extract to remove fatty compounds. The tube content was vortexed for a minute and it was repeated every 5 min. Overall, n-hexane treatment lasted for 20 min. Afterwards, the tubes were placed into the freezer (−18 °C) to remove the residual water and water soluble compounds in the MeCN extract. The ice-cold tubes were then centrifuged (7806 RCF, +4 °C, 10 min); upper n-hexane and bottom water layers were discarded via glass Pasteur pipettes. Ultimately, 1 mL of the MeCN extract was cleaned up using AOAC dSPE kit containing C18 (50 mg), PSA (50 mg), and MgSO_4_ (150 mg). After centrifugation (7806 RCF, +4 °C, 10 min), an aliquot of 500 µL extract was transferred into the LC vials.

##### Extraction of Loin, Content of Caecum, and Hard Feces

Each freeze-dried solid sample (100 mg) was weighed into a 15 mL centrifuge tube. An aliquot of 2.5 µL internal standard (1µg∙mL^−1^) mixture was added onto the sample and mixed. Samples were rehydrated with 1 mL of distilled water and left to stand at 4 °C for 10 min. After rehydration, 1 mL of MeCN was pipetted onto slurry to extract the analytes. Extraction was carried out by using a vortex mixer at 3000 rpm for 20 s. Extraction was repeated three times. At the end of extraction, pre-weighed QuEChERS salt mixture was incorporated into the tube content and briefly vortexed to avoid clumping. An aliquot of 900 µL MeCN layer was removed after centrifugation (7806 RCF, +4 °C, 10 min) and collected in the 2 mL microcentrifuge tubes. In order to remove neutral lipid and fats, 900 µL of n-hexane was incorporated with the sample and mixed continuously on a multi tube vortex mixer for 3 min. The upper hexane layer was discarded with glass Pasteur pipettes after centrifugation and the hexane-treated sample was transferred into the AOAC method dSPE clean-up tubes. After each sample addition, dSPE tubes were mixed and subsequently centrifuged at 7806 RCF rpm at 4 °C for 10 min. An aliquot of 500 µL was transferred into 1.5 mL autosampler vials.

##### Extraction of Blood Samples

An aliquot of 100 µL serum sample was pipetted into a clean 2 mL microcentrifuge tube and mixed with 2.5 µL of internal standard mixture. An aliquot of 1 mL MeCN was transferred onto the sample and whirled continuously on a multi-tube vortex mixer for a minute. After extraction, a pre-weighed 300 mg of QuEChERS salt mixture was incorporated into the sample and the content was shaken vigorously. The MeCN extract was collected after centrifugation and further cleaned up with the prepared dSPE tubes containing C18 (50 mg) and MgSO_4_ (150 mg). The cleaned-up samples were transferred into the LC vials to be analyzed.

##### Extraction of Urine Samples

Urine samples were extracted using the identical QuEChERS method with serum samples. The extract was cleaned up using 25 mg PSA. After clean-up, extracts were transferred to the LC vials.

#### 2.4.4. Analytical Conditions

Residual carbamazepine and metabolites analyses were performed on the ExionLC^TM^ System coupled with ABSciex 6500+ Triple Quadrupole Tandem Mass Spectrometer (AB Sciex, Concord, ON, Canada) with a TurboV source. The injected volume of 2 µL was separated on Ace Excel 2 Super C18 column (100 × 2.1 mm, 3 µm, 90 Å) with a gradient program of mobile phases. The MiliQ water containing 0.1% formic acid and 5 mM ammonium formate was mobile phase A; LC-MS grade methanol was used as mobile phase B. The gradient program (A:B) was started at 40:60 and B concentration reached the 0:100 in 6 min and was maintained for 1 min. Then, mobile phase B concentration was returned to the initial concentration (40:60) at 7.1 min and the column was equilibrated at 40:60 mixture until min 8. Flow rate was set at 0.3 mL∙min^−1^.

The parent drug of CBZ and its metabolites were detected in positive electrospray ionization (ESI+) scheduled multiple reaction monitoring (MRM) mode. Analyte MRM transitions and parameters were listed in Table S1.

Isotopically labelled target analytes (^13^C_6_-CBZ, CBZ-epoxide d_10_) were utilized as internal standards for achieving higher quality quantification. Details of ion source parameters were shown in Table S2. Table S3 presents the limit of quantification (LOQ) results based on the extraction method validation.

### 2.5. Statistical Analysis

Statistical analysis was performed using the computer application SAS 9.4 (SAS Institute, Inc., Cary, NC, USA). One-way analysis of variance (ANOVA) was used. The model used to evaluate all monitored characteristics included the fixed effect of the feed mixture (control, CBZ-low, and CBZ-high dose). The variability inside the tested groups was similar. The significance of the differences between groups was tested by the Duncan’s multiple range test. The value of *p* ≤ 0.05 was considered significant for all measurements. The data are presented as the least square means and their respective standard errors of the means. Means marked with a different superscript letter within each column are significantly different.

## 3. Results

In the present study, the differences between growth and carcass performance; haematological and biochemical parameters; element composition of blood; and content (volume, concentration) of carbamazepine and its metabolites in tissues, internal organs, and body fluids in relation to the carbamazepine addition in growing rabbits were investigated.

### 3.1. Growth and Carcass Performance

The results concerning some growth performance, including live weight at 35, 42, and 77 d of age, body weight gain, and feed intake from 42 to 77 d of age are shown in Table 2. There were no significant differences between the control and experimental groups with CBZ-low and CBZ-high doses.

Some parameters of carcass value and selected physical characteristics in growing rabbits are displayed in Table 3. There were no significant differences between the control and experimental groups, as well as for selected performance parameters.

### 3.2. Haematological and Biochemical Parameters

Selected haematological parameters are presented in Table 4. The values of haematological indicators were within physiological values. All observed characteristics of the blood picture were significantly affected by the addition of CBZ in the feed mixture, with the exception of platelet count. Lower values of selected haematological characteristics were found in the experimental groups with CBZ-low and CBZ-high doses in the feed mixture compared to the control group of rabbits without the addition of CBZ in the feed mixture. Haematological values decreased with increasing doses of CBZ addition in the feed mixture, with the exception of platelet count.

Table 5 shows the biochemical parameter in growing rabbits at the end of the trial. The values of biochemical indicators and element composition were within physiological values. Significant differences were found in total protein, albumin, globulin, alanine aminotransferase, alkaline phosphatase, aspartate aminotransferase, glutamate oxaloacetate transaminase, glutamate pyruvate transaminase, gama glutamyl transferase, urea, triacylglycerol, magnesium, and potassium. Statistically, the highest values of total protein (by 3.92 g·L^−1^; *p* ≤ 0.0047), albumin (by 2.40 g·L^−1^; *p* ≤ 0.0313), and globulin (by 2.31 g·L^−1^; *p* ≤ 0.0224) were found in the blood of experimental rabbits with CBZ-low doses in the feed mixture, while the lowest values were found in the blood of experimental rabbits with CBZ-high doses in the feed mixture. Rabbits that were fed a feed mixture with a CBZ-high dose significantly had the highest values of alanine aminotransferase (by 145.21 IU·L^−1^; *p* ≤ 0.0001), alkaline phosphatase (by 120.79 IU·L^−1^; *p* ≤ 0.0001), aspartate aminotransferase (by 121.72 IU·L^−1^; *p* ≤ 0.0001), glutamate pyruvate transaminase (by 3.01 IU·L^−1^; *p* ≤ 0.0001), gama glutamyl transferase (by 4.52 IU·L^−1^; *p* ≤ 0.0001), urea (by 0.73 IU·L^−1^; *p* ≤ 0.0001), and triacylglycerol (by 0.12 IU·L^−1^; *p* ≤ 0.0001) compared to rabbits from the control group without CBZ. On the other hand, these rabbits (group without CBZ) statistically had the highest value of glutamate oxalocetate transaminase (by 4.49 IU·L^−1^; *p* ≤ 0.0001), amounts of magnesium (by 0.58 mmol·L^−1^; *p* ≤ 0.0001), and potassium (by 0.97 mmol·L^−1^; *p* ≤ 0.0001) in comparison to rabbits with CBZ-high doses. The other elements did not statistically differ among the groups.

### 3.3. Carbamazepine and Its Metabolites in Tissues, Internal Organs, and Body Fluids

CBZ and major metabolite (CBZ-E and CBZ-diol) amounts in solid and liquid samples are shown in Table 6. CBZ, CBZ-E, and CBZ-diol concentration in the monitored samples were significantly affected by the addition of CBZ in the feed mixture, with the exception of the blood sample. It is clear that the concentration of CBZ and its metabolites in samples increases with the amount of carbamazepine in the feed mixture. Some analytes in the control group were also above the limit of quantification, but these are very low and could be either the results of the environmental (the water was tested twice, but its quality cannot be guarantee by scientists) or analytical background.

## 4. Discussion

**Figure 1:**
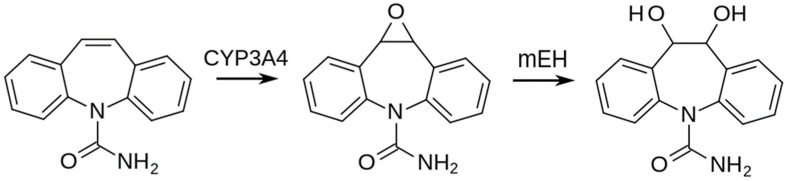
Scheme of metabolism of carbamazepine (carbamazepine; CYP3A4, cytochrome P450 isoenzyme 3A4; carbamazepine-10,11-epoxide, the active metabolite; mEH, microsomal epoxide hydrolase; carbamazepine-10,11-diol, an inactive metabolite).

CBZ (C_15_H_12_N_2_O) is slowly and incompletely absorbed during therapeutic use. One of the primary metabolites of CBZ is carbamazepine-10,11-epoxide (CBZ-E; C_15_H_12_N_2_O_2_), which also has anticonvulsant activity [20]. The CBZ-E is converted stereoselectively (hydrolysis and conjugation) into the corresponding *trans*-10,11-dihydro-10,11-dihydroxy-CBZ (CBZ-diol; C_15_H_14_N_2_O_3_) by epoxide hydrolase [21,22]. CBZ-E is an epoxide and metabolite of CBZ. It has a role as a marine xenobiotic metabolite, a drug metabolite, and an allergen. It is an epoxide, a member of ureas, and a dibenzoazepine. It is functionally related to a carbamazepine [23]. CBZ-diol is a metabolite of the drug CBZ. It has a role as a marine xenobiotic metabolite and a drug metabolite. It is a member of ureas, a diol, and a dibenzoazepine [24]. As described, the main metabolites were found as the most important and thus they were described and discussed in Section 3. Some analytes in the control group were also above the limit of quantification, but these are very low and could be either the results of the environmental (the water was tested twice, but its quality cannot be guarantee by scientists) or analytical background. Nevertheless, the amount of CBZ in the control group was very low and had no potential to compromise the interpretation of the results (Figure 1).

### 4.1. Growth and Carcass Performance

CBZ, phenytoin, valproate, and lamotrigine are typical antiepileptic drugs (AEDs) and are widespread and commonly prescribed to control epileptic seizures. In Europe, CBZ is considered the first-line treatment for epilepsy. The consequence of CBZ usage is that CBZ is ubiquitous in effluents and persistent during wastewater treatment [10]. Some studies [10,13] also detected low levels (ng∙L^−1^) of CBZ in rivers and surface water, and even in drinking water. This is problematic as the AEDs can result in negative adverse events such as gastrointestinal problems, weight gain, or neurologic events, which were found in humans [25]. Increasing body weight is well-known in many AEDs [26,27] due to appetite stimulation and insulin secretion [28]. These findings were reported in humans. In animals, specifically in mammals, information about the effect of CBZ on body parameters is lacking. However, in the study of Zimcikova et al. [7] where rats were exposed to CBZ doses, it was found that the dose of 28 mg∙25 g^−1^ standard laboratory diet for 12 weeks significantly reduced the body weight of the animals compared to the control group with a blood serum drug concentration of 12 µmol∙L^−1^. Nevertheless, the results of the present study did not show any statistical differences among the groups with regard to body weight, weight gain, feed intake, or other productive parameters. Also, the carcass value was not affected by CBZ doses in diet. There is some information about the effect of CBZ on liver regeneration after surgery connected with increased hepatocyte proliferation [29] or on the manifestation of arrhythmias [30], but these are mostly physiological issues with no effect on the weight of the eviscerated organs. It can be assumed that the doses of CBZ which were delivered to rabbits did not influence the productivity or slaughter performance. However, the rabbits had a short fattening period. The doses may be delivered for a longer time to obtain different results as was observed, and longer treatment influenced weight gain in humans [30].

### 4.2. Haematological and Biochemical Parameters

In our study, all haematological parameters were within physiological values, alt-hough CBZ in the feed mixture affected all parameters with the exception of platelet count. Administration of the CBZ in the feed mixture caused a statistically significant decrease in selected haematological parameters compared to the control that received the feed mixture without the addition of CBZ.

Antiepileptic drugs are known to cause a wide range of side effects including haema-tological changes [31]. The changes in haematological biomarkers are extensively used for examining toxic stress and integrity of the immune system [32,33]. CBZ is known to cause a transient reduction in white blood cells during the first 3 months of treatment in humans [34]. This trend was also evident in our experimental model. Leukocytes play a significant role in regulating the immunological function in organisms and changes in the numbers of pollutants, signifying the reduction of non-specific immunity [33]. Red blood cells also showed a downward trend in their number after CBZ administration. It is known that CBZ causes anemia in rats [35,36]. CBZ inhibits colony-stimulating factors in bone marrow [37]. With regard to exposure to CBZ, the hematopoietic centers may have been affected and as a result red and white blood cells reduced. It has also been shown that CBZ can cause thrombocytopenia and can affect the function of platelets and cause prolonged bleeding time in humans [31]. In our study, CBZ did not affect the platelet count in rabbits.

As in the case of haematological parameters, the values of biochemical indicators and element composition were within physiological values. Significant differences were found in total protein, albumin, globulin, alanine aminotransferase, alkaline phosphatase, as-partate aminotransferase, glutamate oxaloacetate transaminase, glutamate pyruvate transaminase, gama glutamyl transferase, urea, triacylglycerol, magnesium, and potassium.

The literature suggests that carbamazepine (CBZ) primarily affects hepatic enzyme activities in human blood serum [38,39]. Elevated levels of these enzymes in the serum can indicate the compromised functional integrity of hepatocytes, leading to the leakage of cellular contents [40]. The increase in alanine aminotransferase, aspartate aminotransferase, gamma-glutamyl transferase, alkaline phosphatase, and other hepatic enzymes indicates hepatocellular damage [41].

Animals exposed to CBZ-low or CBZ-high doses of carbamazepine exhibit elevated levels of urea in their blood serum. Urea, as the primary nitrogenous waste product of protein metabolism, is primarily eliminated from the body through the kidneys via urine [42]. Higher concentrations of urea can serve as an indicator of kidney injury or failure.

Furthermore, the administration of carbamazepine leads to increased levels of triacylglycerol. The alteration in lipid metabolism may be associated with the induction of liver enzymes during carbamazepine treatment and could have clinical implications for the heightened incidence of atherosclerosis and coronary heart disease [43].

Studies have reported that the administration of CBZ results in increased alkaline phosphatase (ALP) activity. This increase may be linked to CBZ’s impact on bone formation, potentially associated with enhanced bone turnover [44].

The CBZ used in our study decreased the level of serum magnesium and potassium in the blood serum of tested rabbits. Anti-epileptic drugs used to control seizures often lead to the depletion of certain minerals (including Mg^2+^). Magnesium has been shown to have an anticonvulsant effect by blocking N-methyl-D-aspartate (NMDA) receptors in neuronal cells. This property is useful in the prevention and management of seizures in pre-eclampsia and eclampsia and control of seizures in epilepsy, hypothyroidism, and glomerulonephritis [45]. When magnesium deficiency is the underlying issue, the seizures are usually resistant to medication and, the longer the patient is not treated with magnesium, the more permanent the damage is [46].

### 4.3. Carbamazepine and Its Metabolites in Tissues, Internal Organs, and Body Fluids

Pharmacokinetic studies examine how a drug is absorbed, metabolized, distributed, and eliminated in the body. CBZ is strongly bound to plasma proteins, specifically protein-bound fractions ranged from 75 to 80%, when the whole plasma was assessed. The main metabolite is CBZ-E [47]. CBZ is almost completely metabolized in the liver (resulting in the 10,11-epoxide metabolite and glucuronides) with approximately 5% of the drug excreted unchanged [48] and therefore higher concentrations of CBZ are found in the liver and kidneys rather than the lungs and brain. The enzyme CYP3A4 plays a crucial role in the metabolism of CBZ by producing the active metabolite CBZ 10,11-epoxide, which is linked to both the toxicity and effectiveness of CBZ [49,50]. In rabbits, carbamazepine metabolism is primarily carried out by CYP3A6, which performs a similar role to CYP3A4 in human hepatocytes. This similarity is due to the comparable p-450 dominance and substrate specificity of the rabbit CYP3A6 and the human CYP3A4 isoform [51]. Additionally, both enzymes are induced by rifampicin [52]. During therapeutic use, the absorption of carbamazepine is slow and incomplete. In cases of large ingestions, absorption can be delayed and unpredictable, resulting in peak levels between 4 and 72 h after the overdose. The absorption phase during an overdose is highly variable due to carbamazepine’s poor solubility, ability to significantly decrease gut motility, and potential for forming pharmacobezoars. The breakdown of carbamazepine produces six other known metabolites, including 10,11-dihydroxycarbamazepine, through further hydrolysis and conjugation. Protein binding is 75% for carbamazepine and 50% for CBZ-E, although this percentage may decrease in cases of a massive overdose due to the saturation of binding sites. The volume of distribution ranges from 0.8 to 1.9 liters per kilogram of body weight. The hydrolyzed and conjugated metabolites are eliminated through the kidneys, with only 1.2% of free carbamazepine found in urine, while 28% is eliminated unchanged through the feces [20], which can be seen in our results in Table 6. Moreover, as shown in Table 6, the amount of CBZ and its metabolites linearly grow with the level of the dose. Most of the unchanged CBZ was found in feces in the CBZ-high group. On the other hand, the lowest amount of CBZ was found in blood as the blood is a pathway of metabolizing CBZ [48]. In the liver and kidneys, a higher amount of CBZ-E than CBZ was found as it is the first metabolite of CBZ and the breakdown is permanent; it is possible to monitor this metabolite from the CBZ of the last days of consumption. It has to be stated that the metabolites or raw CBZ found in tissues, especially in the meat of the loin and thigh, are represented by a relatively low amount when these values are recalculated to mg·kg^−1^ as it is expressed in human doses (200–500 mg·kg^−1^) [53].

## 5. Conclusions

The CBZ doses (CBZ-low and CBZ-high) did not affect growth performance in rabbits. On the other hand, the doses of CBZ caused a decline in the number of leukocytes and other immune-important traits of blood. This was not followed by higher morbidity or mortality but could be challenging in animals with longer fattening periods. In addition, the doses to which the rabbits were exposed mostly left the organism in feces and urine. Minor amounts were detectable in tissues or meat, but the count is not risky in the context of human health when eating this meat.

## Figures and Tables

**Table 1:** Scheme of dividing rabbits into groups and their description.

Group	Group Definition
Control	Feed mixture without CBZ ^1^ ad libitum
CBZ-low dose	0.1 mg CBZ in 10 g of feed mixture per day for 1 kg of live weight + control feed mixture without CBZ ad libitum
CBZ-high dose	12.5 mg CBZ in 10 g of feed mixture per day for 1 kg of live weight + control feed mixture without CBZ ad libitum

^1^ CBZ, carbamazepine (Carbamazepine 5*H*-Dibenz[*b,f*]azepine-5-carboxamide; Merck KGaA, Darmstadt, Germany).

**Table 2:** The effect of CBZ ^1^ addition on growth performance in growing rabbits.

Parameter	Group			SEM ^2^	*p*-Value
	Control	CBZ-Low	CBZ-High		
Live weight 35 d (g)	1031	1027	1051	23.140	0.9065
Live weight 42 d (g)	1500	1491	1506	29.669	0.9806
Live weight 77 d (g)	3136	3013	3006	79.617	0.6831
Period 42–77 d					
Daily weight gain (g)	47.64	46.60	44.62	1.484	0.7087
Total weight gain (g)	1668	1527	1514	52.629	0.4360
Daily feed intake (g)	185.58	183.56	172.11	6.400	0.6708
Feed conversion ratio	4.20	3.99	4.23	0.109	0.6548

^1^ CBZ, carbamazepine; ^2^ SEM, standard error of the mean.

**Table 3:** The effect of CBZ ^1^ addition on some parameters of carcass value and selected physical characteristics in growing rabbits.

Parameter	Group			SEM ^2^	*p*-Value
	Control	CBZ-Low	CBZ-High		
Slaughter weight (g)	3163	3013	3006	79.617	0.6831
Hot carcass (g)	1946	1908	1816	54.631	0.6259
Chilled carcass (g)	1888	1850	1755	53.535	0.6012
Reference carcass (g)	1559	1527	1441	42.311	0.5189
Cooler carcass shrinkage (%)	3.01	3.08	3.31	6.181	0.7952
Dressing percentage (%)	59.65	61.77	58.43	1.160	0.5150
Liver (% CC ^3^)	5.89	5.98	5.79	0.193	0.9283
Kidney (% CC)	1.06	1.06	1.15	0.034	0.4766
Hind leg (% CC)	29.97	29.71	29.41	0.3143	0.7823
pHu ^4^ of the loin	5.43	5.40	5.35	0.013	0.0595
pHu of the thigh	5.56	5.54	5.57	0.017	0.6746

^1^ CBZ, carbamazepine; ^2^ SEM, standard error of the mean; ^3^ CC, chilled carcass; ^4^ pHu, pH 24 h post mortem.

**Table 4:** The effect of CBZ ^1^ addition on selected parameters of blood picture in growing rabbits at 77 d of age.

Parameter	Group			SEM ^2^	*p*-Value
	Control	CBZ-Low	CBZ-High		
Haematocrit (%)	48.96 ^a^	42.56 ^b^	42.83 ^b^	0.692	0.0001
Haemoglobin (g·L^−1^)	130.69 ^a^	112.81 ^b^	113.84 ^b^	1.882	0.0001
Erythrocytes (T·L^−1^)	6.31 ^a^	5.49 ^b^	5.50 ^b^	0.090	0.0001
Leukocytes (G·L^−1^)	10.82 ^a^	9.97 ^b^	9.58 ^c^	0.130	0.0001
Neutrophils (G·L^−1^)	5.41 ^a^	4.99 ^b^	4.79 ^c^	0.065	0.0001
Lymphocytes (G·L^−1^)	3.46 ^a^	3.19 ^b^	3.06 ^c^	0.042	0.0001
Monocytes (G·L^−1^)	1.41 ^a^	1.30 ^b^	1.24 ^c^	0.017	0.0001
Eosinophils (G·L^−1^)	0.43 ^a^	0.40 ^b^	0.38 ^b^	0.005	0.0001
Basophils (G·L^−1^)	0.11 ^a^	0.10 ^b^	0.10 ^b^	0.002	0.0001
Platelet count (G·L^−1^)	550.93	548.30	568.54	8.049	0.5571

^1^ CBZ, carbamazepine; ^2^ SEM, standard error of the mean; ^abc^ data bearing different letters in the same row are significantly different (*p* ≤ 0.05).

**Table 5:** The effect of CBZ ^1^ addition on some biochemical parameters and element composition in growing rabbits at 77 d of age.

Parameter	Group			SEM ^2^	*p*-Value
	Control	CBZ-Low	CBZ-High		
Total protein (g·L^−1^)	61.91 ^b^	64.71 ^a^	60.79 ^b^	0.543	0.0047
Albumin (g·L^−1^)	33.91 ^ab^	35.11 ^a^	32.71 ^b^	0.386	0.0313
Globulin (g·L^−1^)	33.18 ^ab^	34.75 ^a^	32.39 ^b^	0.371	0.0224
Albumin/globulin	1.02	1.01	1.01	0.007	0.8083
Alanin aminotransferase (IU·L^−1^)	167.19 ^c^	274.32 ^b^	312.40 ^a^	12.882	0.0001
Alkaline phosphatase (IU·L^−1^)	54.06 ^c^	118.87 ^b^	174.85 ^a^	10.490	0.0001
Amylase (IU·L^−1^)	308.83	407.25	363.79	21.298	0.1697
Aspartate aminotransferase (IU·L^−1^)	41.15 ^c^	107.28 ^b^	162.87 ^a^	11.035	0.0001
Glutamate oxalocetate transaminase (IU·L^−1^)	57.29 ^a^	53.82 ^b^	52.71 ^c^	0.451	0.0001
Glutamate pyruvate transaminase (IU·L^−1^)	93.41 ^c^	94.73 ^b^	96.42 ^a^	0.298	0.0001
Gama glutamyl transferase (IU·L^−1^)	11.85 ^c^	15.26 ^b^	16.37 ^a^	0.426	0.0001
Kreatinin (mmol·L^−1^)	78.51	77.89	72.27	4.524	0.8377
Glucose (mmol·L^−1^)	6.48	5.71	6.12	0.311	0.6173
Urea (mmol·L^−1^)	7.67 ^b^	8.31 ^a^	8.40 ^a^	0.074	0.0001
Triacylglycerol (mmol·L^−1^)	0.85 ^b^	0.95 ^a^	0.97 ^a^	0.011	0.0001
Cholesterol (mmol·L^−1^)	1.30	1.46	1.04	0.119	0.3468
Calcium (mmol·L^−1^)	4.26	4.30	4.05	0.124	0.6969
Phosphorus (mmol·L^−1^)	5.03	4.80	4.91	0.119	0.7624
Chlorine (mmol·L^−1^)	112.73	112.92	110.64	0.977	0.5948
Magnesium (mmol·L^−1^)	1.10 ^a^	0.56 ^b^	0.52 ^b^	0.060	0.0001
Potassium (mmol·L^−1^)	3.98 ^a^	3.20 ^b^	3.01 ^b^	0.114	0.0001
Sodium (mmol·L^−1^)	143.11	143.28	140.90	0.834	0.4511

^1^ CBZ, carbamazepine; ^2^ SEM, standard error of the mean; ^abc^ data bearing different letters in the same row are significantly different (*p* ≤ 0.05).

**Table 6:** The effect of CBZ ^1^ addition on CBZ and major metabolite distribution in solid tissue and liquid samples in growing rabbits at 77 d of age (ppb).

Sample	Compound/ Metabolite	Group			SEM ^2^	*p*-Value
		Control	CBZ-Low	CBZ-High		
Liver	CBZ ^1^	3.0 ^b^	21.0 ^b^	624.6 ^a^	73.982	0.0001
	CBZ-E ^3^	0.3 ^b^	22.2 ^b^	1360.8 ^a^	165.058	0.0001
	CBZ-diol ^4^	0.08 ^b^	4.22 ^b^	248.70 ^a^	27.102	0.0001
Spleen	CBZ	0 ^b^	5.98 ^b^	193.21 ^a^	23.759	0.0001
	CBZ-E	0 ^b^	9.0 ^b^	661.2 ^a^	88.307	0.0003
	CBZ-diol	0.016 ^b^	1.258 ^b^	67.268 ^a^	7.103	0.0001
Kidney	CBZ	0.39 ^b^	80.77 ^b^	261.87 ^a^	34.22	0.0021
	CBZ-E	0.1 ^b^	95.5 ^b^	1060.6 ^a^	119.988	0.0001
	CBZ-diol	0.17 ^b^	7.10 ^b^	223.26 ^a^	22.935	0.0001
Caecum	CBZ	0.3 ^b^	94.1 ^b^	2026.2 ^a^	203.827	0.0001
	CBZ-E	0 ^b^	32.3 ^b^	2179.1 ^a^	239.855	0.0001
	CBZ-diol	0.5 ^b^	33.4 ^b^	1800.6 ^a^	217.328	0.0001
Feces	CBZ	0.8 ^b^	209.7 ^b^	5507.6 ^a^	693.702	0.0001
	CBZ-E	0 ^b^	72.3 ^b^	2692.7 ^a^	381.444	0.0001
	CBZ-diol	0 ^b^	15.2 ^b^	660.0 ^a^	120.927	0.0032
Urine	CBZ	1.68 ^b^	6.69 ^b^	77.96 ^a^	11.670	0.0058
	CBZ-E	2.56 ^b^	17.48 ^b^	448.13 ^a^	51.797	0.0001
	CBZ-diol	2.31 ^b^	12.28 ^b^	163.15 ^a^	33.465	0.0002
Blood	CBZ	0.0304	0.0598	0.1075	0.0145	0.0842
	CBZ-E	0	0	0.0007	0.0002	0.2760
	CBZ-diol	0.0373	0.0360	0.0359	0.0023	0.9643
Loin meat	CBZ	0.75 ^b^	8.66 ^b^	188.88 ^a^	22.957	0.0001
	CBZ-E	0 ^b^	8.4 ^b^	684.3 ^a^	88.971	0.0002
	CBZ-diol	0.14 ^b^	1.64 ^b^	135.03 ^a^	14.363	0.0001
Thigh meat	CBZ	0.15 ^b^	9.10 ^b^	184.96 ^a^	21.823	0.0001
	CBZ-E	0 ^b^	10.9 ^b^	796.4 ^a^	101.183	0.0001
	CBZ-diol	0.02 ^b^	2.52 ^b^	168.70 ^a^	17.892	0.0001

^1^ CBZ, carbamazepine; ^2^ SEM, standard error of the mean; ^3^ CBZ-E, CBZ-10,11-epoxide; ^4^ CBZ-diol, *trans*-10,11-dihydro-10,11-dihydroxy-CBZ; ^ab^ data bearing different letters in the same row are significantly different (*p* ≤ 0.05).

## Data Availability

The data presented in this study are available upon reasonable request from the corresponding author.

## Supplementary Materials

The following supporting information can be downloaded at: https://www.mdpi.com/article/10.3390/ani13122041/s1, Table S1. List of analytes with MRM transitions and parameters. Table S2. Ion source parameters-Electrospray ionization conducted in positive mode. Table S3. Limit of quantification (LOQ) results based on the extraction method validation.
