# Supplementary material for: The Effect of Carbamazepine on Performance, Carcass Value, Hematological and Biochemical Blood Parameters, and Detection of Carbamazepine and Its Metabolites in Tissues, Internal Organs, and Body Fluids in Growing Rabbits
Source: Animals (Basel). 2023 Jun 20;13(12):2041. doi: 10.3390/ani13122041 (PMC10295208; doi:10.3390/ani13122041)
Supplement: Supplementary file 1 [file animals-13-02041-s001.zip › animals-2419694-supplementary.pdf]

animals MDPI

Table S1. List of analytes with MRM [1] transitions and parameters.

Analyte	Q1 [2] ($m \cdot z^{-1}$)	Q3 [3] ($m \cdot z^{-1}$)	RT [4] (min)
CBZ-1 [5]	237.06	194	2.12
CBZ-2 [6]	237.06	165	2.12
CBZ-epoxide-1	253.045	179.9	1.43
CBZ-epoxide-2	253.045	236.1	1.43
trans-CBZ diol-1	271.04	210	1.31
trans-CBZ diol-2	271.04	180.1	1.31
$^{13}C_6$-CBZ-ISTD [7]	243.098	200	2.11
CBZ-epoxide-d_{10}-ISTD [8]	263.099	246.1	1.41

[1] MRM, Multiple Reaction Monitoring; [2] Q1, $M + H^+$; [3] Q3, fragment ion; [4] RT, retention time (min); [5] CBZ-1, carbamazepine-1 quantifier ion; [6] CBZ-2, carbamazepine-2 quantifier ion; [7] $^{13}C_6$-CBZ-ISTD, $^{13}C_6$-CBZ internal standards; [8] CBZ-epoxide-d_{10}-ISTD, CBZ-epoxide-d_{10} internal standards.

Table S2. Ion source parameters—Electrospray ionization conducted in positive mode.

Parameter	Setting
Curtain Gas	35 psi
Collision Gas	Medium
IonSpray Voltage	5500 V
Temperature	550 °C
Ion Source Gas 1	40 psi
Ion Source Gas 2	50 psi

Table S3. Limit of quantification (LOQ) results based on the extraction method validation.

Analysed Group	Sampling Weight/Volume	CBZ [1]	CBZ-E [2]	CBZ-diol [3]
Urine	0.10 mL	1 [4]	0.5	0.5
Feces	0.10 g	10	0.5	0.5
Content of Caecum	0.10 g	1	0.5	10
Serum/Plasma	0.10 mL	10	0.5	n.v [5]
Liver	0.10 g	0.5	0.5	1
Kidney	1.00 g	25	1	1
Meat of loin	1.00 g	0.5	0.5	1
Meat of thigh	0.10 g	0.5	0.5	10
Spleen	0.10 g	1	1	n.v

[1] CBZ, carbamazepine; [2] CBZ-E, CBZ-10,11-epoxide; [3] CBZ-diol, *trans*-10,11-dihydro-10,11-dihydroxy-CBZ; [4] Limit of quantification at $ng \cdot g^{-1}$ (ppb); [5] n.v, not validated.
